# Site-specific mutagenesis of *Drosophila *proliferating cell nuclear antigen enhances its effects on calf thymus DNA polymerase δ

**DOI:** 10.1186/1471-2091-5-13

**Published:** 2004-08-13

**Authors:** Dmitry Ju Mozzherin, Maeve McConnell, Holly Miller, Paul A Fisher

**Affiliations:** 1 The Department of Pharmacological Sciences University Medical Center State University of New York at Stony Brook Stony Brook, NY 11794-8651 USA; 2 The Department of Pharmacological Sciences Laboratory of Chemical Biology University Medical Center State University of New York at Stony Brook Stony Brook, NY 11794-8651 USA

## Abstract

**Background:**

We and others have shown four distinct and presumably related effects of mammalian proliferating cell nuclear antigen (PCNA) on DNA synthesis catalyzed by mammalian DNA polymerase δ(pol δ). In the presence of homologous PCNA, pol δ exhibits 1) increased absolute activity; 2) increased processivity of DNA synthesis; 3) stable binding of synthetic oligonucleotide template-primers (t_1/2 _of the pol δ•PCNA•template-primer complex ≥2.5 h); and 4) enhanced synthesis of DNA opposite and beyond template base lesions. This last effect is potentially mutagenic *in vivo*. Biochemical studies performed in parallel with *in vivo *genetic analyses, would represent an extremely powerful approach to investigate further, both DNA replication and repair in eukaryotes.

**Results:**

*Drosophila *PCNA, although highly similar in structure to mammalian PCNA (e.g., it is >70% identical to human PCNA in amino acid sequence), can only substitute poorly for either calf thymus or human PCNA (~10% as well) in affecting calf thymus pol δ. However, by mutating one or only a few amino acids in the region of *Drosophila *PCNA thought to interact with pol δ, all four effects can be enhanced dramatically.

**Conclusions:**

Our results therefore suggest that all four above effects depend at least in part on the PCNA-pol δ interaction. Moreover unlike mammals, *Drosophila *offers the potential for immediate *in vivo *genetic analyses. Although it has proven difficult to obtain sufficient amounts of homologous pol δ for parallel *in vitro *biochemical studies, by altering *Drosophila *PCNA using site-directed mutagenesis as suggested by our results, *in vitro *biochemical studies may now be performed using human and/or calf thymus pol δ preparations.

## Background

Many *Drosophila melanogaster *homologs of the proteins required for both DNA replication and repair have been identified and in several cases purified to apparent homogeneity. These include DNA polymerase α holoenzyme [1,2], DNA polymerase δ(pol δ) [2-4], replication protein A (RP-A; [5]), replication factor C (RF-C; e.g., see [6-9]) and various origin recognition complex (ORC) subunits (see e.g., [10,11]). Moreover, complete replication of DNA containing the SV40 origin of replication has been reconstituted *in vitro *using purified SV40 T-antigen and *Drosophila *cell-free extracts [7].

A protein about which much information has been obtained is proliferating cell nuclear antigen (PCNA). *Drosophila *PCNA was first identified both as a highly purified protein able to substitute, albeit poorly, for human PCNA in a cell-free SV40 DNA replication system reconstituted from purified proteins [12] and by Yamaguchi et al. [13] who used an oligonucleotide probe to detect the *Drosophila *PCNA cDNA and gene, express the protein in *E. coli *and deduce its complete amino acid sequence. Further results indicated that in flies, PCNA was encoded by a single gene located at position 56F5-15 on the right arm of chromosome 2. This was subsequently identified as the *Drosophila mus*209 locus [14]. Recently, a second *Drosophila *PCNA gene of limited homology to the original and of unknown biological function has also been found [15].

Protocols have been established for purification of wild-type human PCNA from tissue culture cells [16,17], unmodified wild-type human PCNA after regulated expression in *E. coli *[18] and NH_2_-terminally his-tagged but otherwise wild-type human PCNA, also engineered for bacterial expression [19]. All were comparably effective at stimulating mammalian pol δ. Similar protocols have been developed for *Drosophila *PCNA and strategies for site-directed mutagenesis have been devised and implemented [20].

Recently, Zhang et al. [21] (see also [22]) as well as others (e.g., see [23]) identified the interdomain connector loop of PCNA (amino acids 119-133 of human PCNA) as crucial for binding pol δ. Of note, relative to wild-type PCNA, mutations of the molecule within this region such as glutamine at position 125 changed to glutamic acid (Q125E) promoted increased pol δ-processivity [21]. In human PCNA, residues 123, 126, 127 and 128 were defined as being essential for interaction with pol δ [21]. Comparison of human with *Drosophila *PCNA sequences in this region indicated that of these four amino acids, three (residues 126, 127 and 128) are identical. The fourth, residue 123, is glutamine (Q123) in wild-type *Drosophila *PCNA. The corresponding residue in human PCNA is valine (V).

To investigate the role of the interdomain connector loop of PCNA on the effects of PCNA on pol δ, we mutagenized residues within this region of *Drosophila *PCNA so that they more nearly resembled human amino acids. After bacterial expression and purification, we tested the effects of these site-specifically modified ("humanized") *Drosophila *PCNA molecules on purified calf thymus pol δ (two-subunit form; see [17,24]). Calf thymus and human pol δ are highly similar in amino acid sequence [25-27] and can, for our purposes, be used interchangeably. "Humanization" of a single *Drosophila *PCNA residue, conversion of Q123 to V (Q123V), conferred upon it, enhanced ability to affect several properties of calf thymus pol δ. More extensive mutagenesis, in which the entire interdomain connector loop of *Drosophila *PCNA (amino acids 119-133) was replaced by the corresponding human residues, was still more effective at stimulation of calf thymus pol δ, than either wild-type or Q123V *Drosophila *PCNA. However, it was considerably less effective than wild-type human PCNA at altering the properties of calf thymus pol δ. These results therefore suggest that in addition to the interdomain connnector loop, other regions of PCNA are also important effectors of pol δ activity. They also provide a means to couple operationally, the considerable power of *in vivo *genetic analyses performed in *Drosophila *with the sophistication of mammalian biochemistry.

## Results

**Figure 1:**
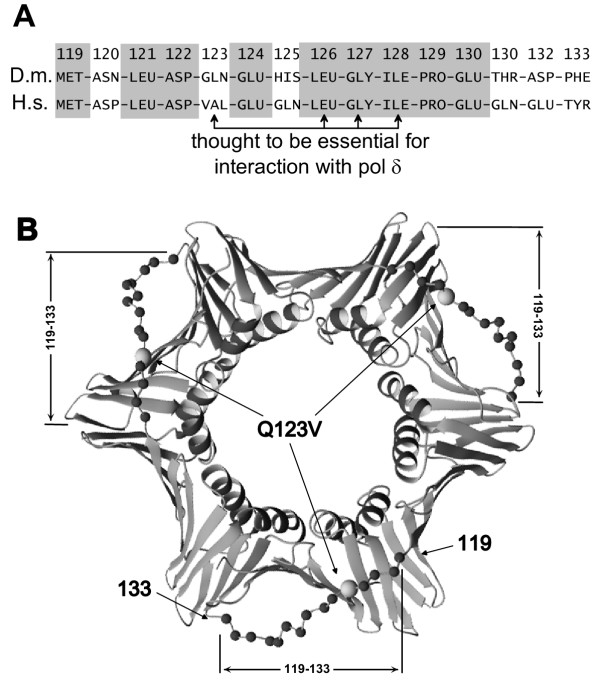
**Structure and structural rationale for mutating *Drosophila *PCNA. **A: amino acid sequences of the interdomain loops of *Drosophila *(designated D.m.) and human (designated H.s.) PCNA. Gray boxes indicate amino acids identical for both organisms; arrows show amino acids thought essential for interaction of human PCNA with human pol δ. Amino acid 123 is the only one which is both essential and different in *Drosophila *versus human PCNA. B: the "front" side of the human PCNA trimer. Amino acids 119-133 of the interdomain loops are highlighted by showing their α-carbon atoms as black spheres. The α-carbon atom of Val123 is shown as a larger gray sphere.

To study the role of the interdomain connector loop of PCNA (amino acids 119-133), we compared human and *Drosophila *homologs. Of the 15 interdomain connector loop residues, nine are identical between the two; identical residues are shaded (Fig. 1A). Overall, *Drosophila *PCNA is >70% identical to that from mammals (e.g., humans; see [13]). Others showed that PCNA residues 123, 126, 127 and 128 were essential for interaction with pol δ [28]. Of these four, only one (residue 123) differs between flies and humans. Also shown is a model constructed from the X-ray crystallographically determined structure of PCNA indicating the locations of the sites to be mutated in *Drosophila *PCNA (Fig. 1B). Shown (Fig. 1B) is the X-ray crystal structure of human PCNA. The *Drosophila *homolog is assumed to be similar.

### Purification of wild-type and site-specifically mutated PCNA

**Figure 2:**
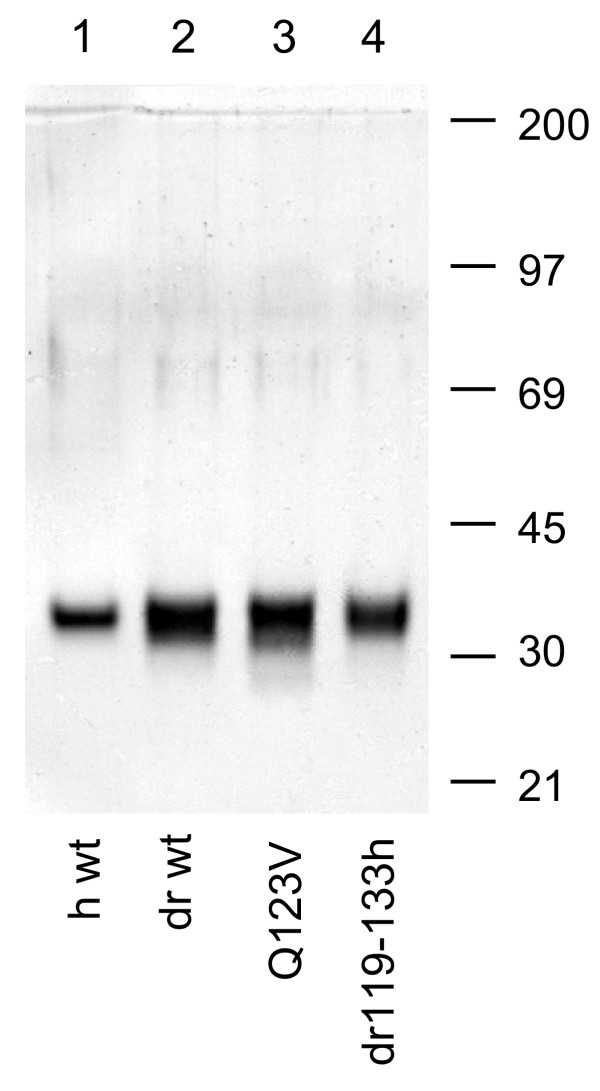
**SDS-PAGE analysis of his-tagged PCNA purified from *E. coli *extracts after regulated bacterial expression. **Purification and SDS-PAGE were as described (Experimental Procedures). Lane 1, 0.4 μg wild-type human PCNA was subjected to electrophoresis. Lane 2, 0.8 μg wild-type *Drosophila *PCNA was subjected to electrophoresis. Lane 3, 0.8 μg *Drosophila *PCNA containing valine substituted for glutamine at position 123 was subjected to electrophoresis. Lane 4, 0.45 μg *Drosophila *PCNA containing amino acids 119-133 substituted with the corresponding human PCNA amino acids was subjected to electrophoresis. Migration positions of molecular mass standards are indicated to the right of the figure.

Four NH_2_-terminally his-tagged PCNA variants were highly purified; purity for each is shown (Fig. 2). First constructs were prepared encoding 1) NH_2_-terminally his-tagged wild-type human PCNA; 2) NH_2_-terminally his-tagged wild-type *Drosophila *PCNA (dPCNA) and two dPCNA derivatives; 3) one in which amino acid 123 was mutated from glutamine to valine (Q123V dPCNA); and 4) the other, in which *Drosophila *amino acids 119-133 were replaced by the corresponding human sequence (dr119-133h dPCNA). Then all four were transformed separately into *E. coli *(strain M15 [pREP4]) and respective proteins were expressed. Finally bacteria were lysed and his-tagged proteins were purified using various procedures including Ni^2+^-IDA Sepharose chromatography. The purity of each was determined by SDS-PAGE and is shown as indicated (Fig. 2). The identity of wild-type human PCNA was confirmed using mouse monoclonal anti-mammalian PCNA antibody PC10; the identity of wild-type *Drosophila *PCNA was confirmed using affinity purified polyclonal anti-*Drosophila *PCNA antibodies prepared in rabbits [12] (not shown).

### Stimulation of calf thymus pol δ activity by highly purified wild-type versus selected mutant PCNA fractions

**Figure 3:**
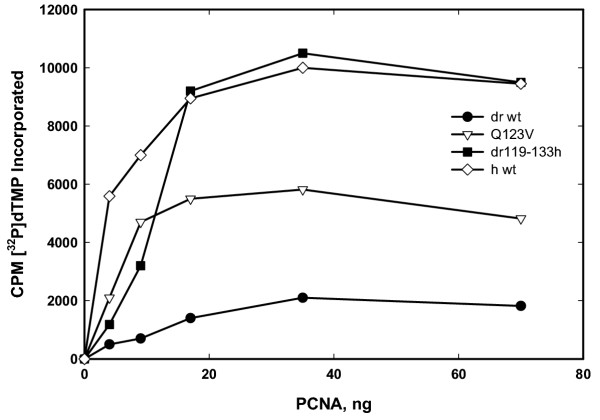
**Effect of various purified PCNA fractions on the DNA polymerase activity of calf thymus pol δ. **Calf thymus pol δ was incubated in a reaction mixture as described (see Materials and Methods) for 5 min at room temperature. Each incubation contained 10 ng of pol δ. DNA product synthesized was determined after placing 5-μl aliquots on Whatman DE-81 filters and subsequently washing with a 5% (w/v) solution of Na_2_HPO_4_•12H_2_O. Radioactivity retained on filters was then determined by liquid scintillation counter. Reaction mixtures contained increasing amounts, as indicated on the abscissa, of various PCNA samples, also as indicated.

Calf thymus pol δ (apparently homogeneous two-subunit form; see [24]) was purified and assayed for polymerase activity in the presence of varying concentrations of both highly purified wild-type and specific mutant PCNA molecules. We showed previously that either calf thymus or human PCNA could be used interchangeably as stimulatory co-factors for calf thymus pol δ [29] (see also [12,18,19]). Assays were performed using poly(dA)-oligo(dT) as described (Experimental Procedures). As can be seen, human PCNA resulted in robust stimulation of calf thymus pol δ; much less stimulation was observed for wild-type *Drosophila *PCNA (Fig. 3). Mutation of *Drosophila *PCNA resulted in substantially increased stimulation of calf thymus pol δ; both substitution of a single amino acid (Q123V dPCNA) and replacement of the entire fly interdomain connector loop with corresponding human amino acids (dr119-133h dPCNA) had demonstrable effects. Of note, at relatively high concentrations, *Drosophila *PCNA but with the entire fly interdomain connector loop replaced by corresponding human amino acids (dr119-133h dPCNA) was similarly effective to wild-type human PCNA at stimulating the activity of calf thymus pol δ; however, it was considerably less effective at lower concentrations (Fig. 3). This suggests an effect on binding of PCNA to pol δ and/or on mutant PCNA multimerization.

### The effects of highly purified wild-type versus selected mutant PCNA fractions on the processivity of incorporation by calf thymus pol δ

**Figure 4:**
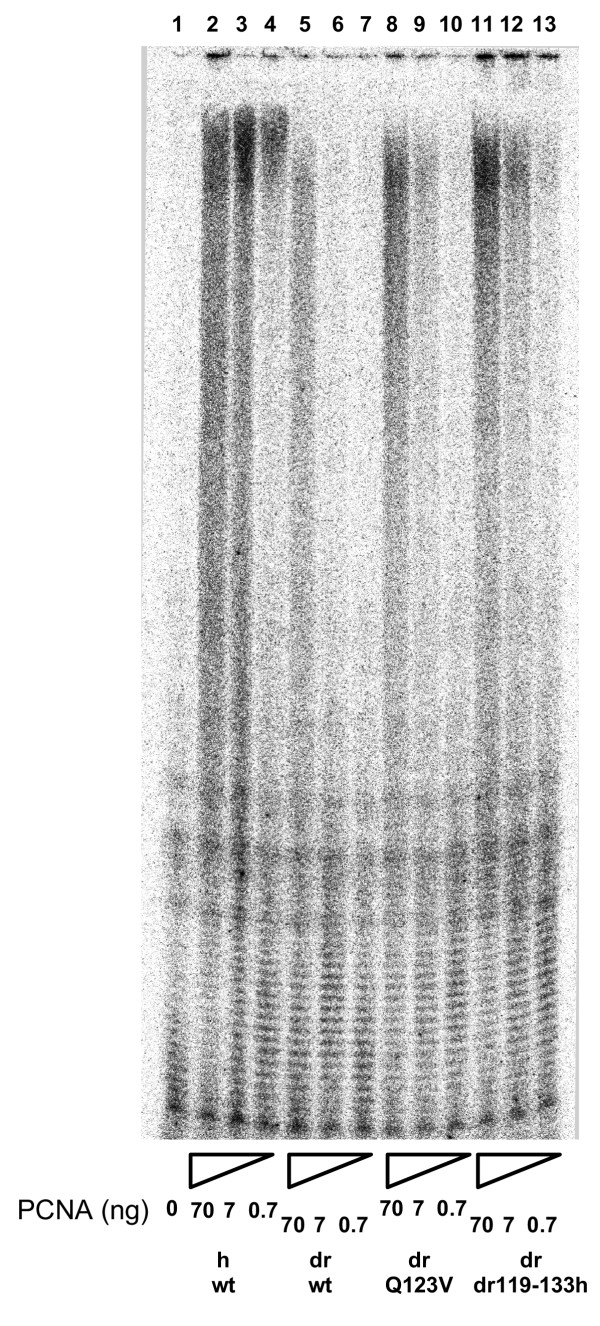
**Effect of various purified PCNA fractions on the processivity of nucleotide incorporation by calf thymus pol δ. **Incorporation of [α^32^P]dTMP by calf thymus pol δ was monitored by standard denaturing PAGE. The substrates used were (dA)_~500_-(dT)_12–18 _as template-primer and [α-^32^P]dTTP. Concentrations of PCNA, both wild-type and mutant proteins, are as indicated. h, human; dr, *Drosophila melanogaster. *NH_2_-terminally his-tagged-PCNA fractions are as indicated; wt, wild-type; Q123V, recombinant *Drosophila *PCNA containing a single amino acid, glutamine at position 123, changed to valine; dr119-133h, recombinant *Drosophila *PCNA containing the entire interdomain connector loop (amino acids 119-133) replaced with the corresponding human PCNA amino acids.

To examine further, the stimulation of calf thymus pol δ by both wild-type and specific mutant PCNA molecules, we examined effects on processivity of nucleotide incorporation. Processivity is defined as the number of deoxyribonucleotides incorporated each time a DNA polymerase binds its template-primer. As can be seen, without PCNA (Fig. 4 lane 1), pol δ is essentially a distributive enzyme incorporating only a few nucleotides as a result of each binding event. With increasing concentrations of wild-type human PCNA (concentrations increasing from right to left as indicated), processivity of incorporation increases dramatically (Fig. 4 lanes 2–4). This correlates quite closely with the PCNA-mediated activity increase (see Fig. 3). Wild-type *Drosophila *PCNA had relatively much less effect on the processivity of calf thymus pol δ (Fig. 4 lanes 5–7; concentrations again increasing from right to left as indicated). This is also consistent with activity data presented herein (Fig. 3) as well as with results reported previously [12]. When mutants of *Drosophila *PCNA were tested, both Q123V dPCNA (Fig. 4 lanes lanes 8–10; concentrations again increasing from right to left as indicated) and dr119-133h dPCNA (Fig. 4 lanes lanes 11–13; concentrations again increasing from right to left as indicated), promoted increased pol δ processivities, again consistent with increased activities (Fig. 3). Increases were concentration-dependent, also as expected.

### Stable complex formation among pol δ, ^32^P-labeled oligonucleotide template-primer and highly purified wild-type versus selected mutant PCNA fractions

**Figure 5:**
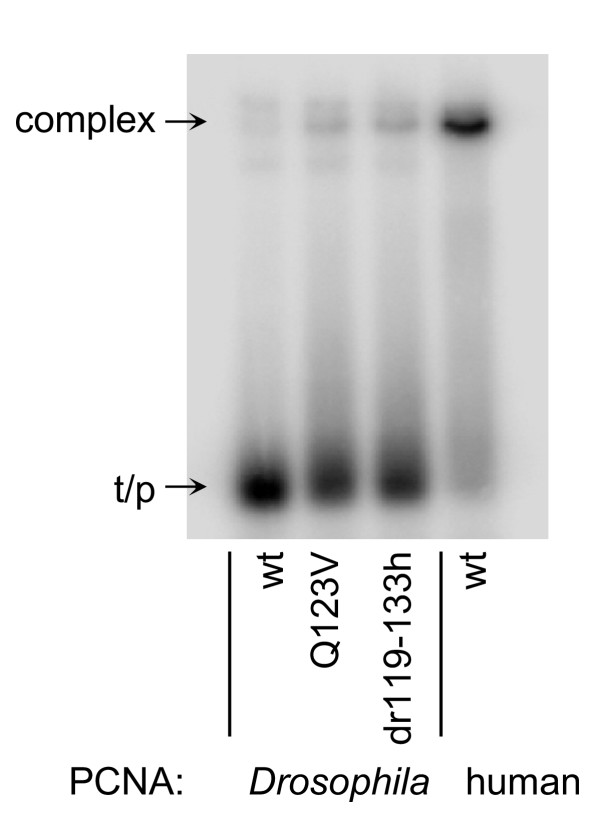
**Effect of various purified PCNA fractions on calf thymus pol δ•PCNA•^32^P-labeled oligonucleotide template-primer complex formation. **Complex formation among pol δ, various purified PCNA fractions and ^32^P-labeled synthetic oligonucleotide template-primers (30-21-mers) was monitored by standard non-denaturing PAGE-band-mobility-shift assays [32]. Each incubation contained 10 ng of pol δ, 70 ng of PCNA and 0.1 pmol/reaction (useable 3'-OH) of annealed template-primer. NH_2_-terminally his-tagged-PCNA fractions are as indicated; wt, wild-type; Q123V, recombinant *Drosophila *PCNA containing a single amino acid, glutamine at position 123, changed to valine; dr119-133h, recombinant *Drosophila *PCNA containing the entire interdomain connector loop (amino acids 119-133) replaced with the corresponding human PCNA amino acids.

PAGE band mobility shift assays were used to evaluate, in an essentially qualitative manner, the stability of complex formation among calf thymus pol δ, labeled template-primer and highly purified wild-type versus selected mutant PCNA molecules. As can be seen, wild-type *Drosophila *PCNA promoted almost no pol δ•PCNA•template-primer complex formation (Fig. 5). In contrast, complex-formation with both *Drosophila *PCNA mutants (Q123V dPCNA and dr119-133h dPCNA) was readily detectable but neither gave results as robust as those seen with wild-type human PCNA (Fig. 5).

### DNA synthesis beyond chemically defined template base lesions promoted by highly purified wild-type versus selected mutant PCNA fractions

**Figure 6:**
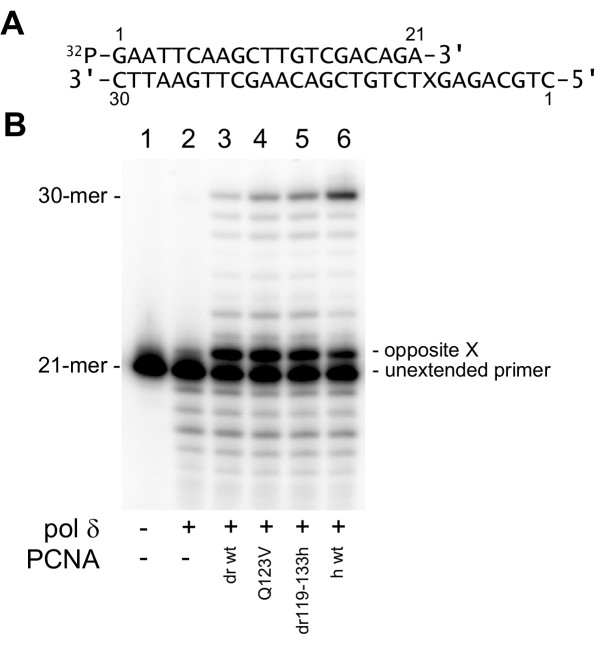
**Effect of various purified PCNA fractions to promote nucleotide incorporation by calf thymus pol δ beyond chemically defined template base lesions. **A: the structure of the 5'-^32^P-labeled 30-21-mer template-primer; only the primer (21-mer) was radiolabeled and X indicates the position of a modified tetrahydrofuran moiety (model abasic site) on the 30-mer template. B: lane 1, gel-purified primer alone was subjected to electrophoresis; lanes 2–6, incubations were formulated as indicated with the template-primer shown in A followed by standard denaturing PAGE. h, human; dr, *Drosophila melanogaster. *For lanes 2–6, each incubation contained 0.5 pmol of labeled primer (3'-OH ends) annealed to 0.5 pmol of template (3'-OH ends), 10 ng pol δ and 70 ng PCNA as indicated. NH_2_-terminally his-tagged-PCNA fractions are as indicated; wt, wild-type; Q123V, recombinant *Drosophila *PCNA containing a single amino acid, glutamine at position 123, changed to valine; dr119-133h, recombinant *Drosophila *PCNA containing the entire interdomain connector loop (amino acids 119-133) replaced with the corresponding human PCNA amino acids.

As a final test, we examined the abilities of various PCNA fractions to promote pol δ-dependent DNA synthesis beyond template base lesions (TLS). PCNA-dependent TLS by pol δ was first reported by O'Day et al. [30] and subsequently analyzed in detail biochemically [29]. The structure of the synthetic oligonucleotide used for evaluation is shown in Fig. 6A. For the data shown (Fig. 6B), *X *represents the model abasic site (hereafter termed the abasic site [31]) used previously for many of our studies (e.g., see [29]). The mobility of the labeled 21-mer primer, PAGE-purified but without any subsequent enzymatic incubation is shown (Fig. 6B lane 1). When calf thymus pol δ alone was added, primer extension opposite the template abasic site was detected but there was no discernible elongation of the resulting 22-mer primer and no full-length product (30-mer) was observed; some degradation of the 21-mer primer, presumably resulting from the activity of the intrinsic pol δ 3'-5' exonuclease, was seen (Fig. 6B lane 2). Addition to incubations of wild-type *Drosophila *PCNA resulted in slight but readily detectable DNA synthesis beyond the template abasic site; this included some full-length 30-mer (Fig. 6B lane 3). Relatively more full-length 30-mer was seen when Q123V mutant *Drosophila *PCNA was included in addition to calf thymus pol δ (Fig. 6B lane 4) and still more full-length 30-mer was seen when dr119-133h *Drosophila *PCNA was added (Fig. 6B lane 5). Clearly, the greatest amount of full-length 30-mer product was seen when wild-type human PCNA was incubated with calf thymus pol δ (Fig. 6B lane 6). Of note, wild-type human PCNA also promotes the tightest complex formation between calf thymus pol δ and ^32^P-labeled template-primer DNA (see Fig. 5).

## Discussion

Although human PCNA and *Drosophila *PCNA are more than 70% identical at the level of primary amino acid sequence, wild-type *Drosophila *PCNA is only a very poor substitute for human PCNA in cell-free reactions with calf thymus pol δ. This is documented both in this report and previously [12,32]. However, mutating only a single *Drosophila *PCNA amino acid, glutamine at position 123 (Q123) to valine (V), leads to a dramatic enhancement in the abilities of *Drosophila *PCNA to stimulate calf thymus pol δ. Effects were shown on total activity (Fig. 3), processivity (Fig. 4), pol δ•PCNA•template-primer complex formation (Fig. 5) and extended DNA synthesis beyond a template abasic site (Fig. 6). Replacing the entire interdomain connector loop of *Drosophila *PCNA (amino acids 119-133) with the corresponding residues from human PCNA resulted in additional enhancement (Figs. 3,4,5,6), but in neither case were the mutants of *Drosophila *PCNA (Q123V dPCNA or dr119-133h dPCNA) equivalent to wild-type human PCNA in the stimulation of calf thymus pol δ.

Our data indicate that although a single *Drosophila *PCNA amino acid at position 123 (in addition to conserved residues 126–128) is very important for pol δ-stimulation, the further enhancement of stimulation seen when the entire interdomain connector loop of *Drosophila *PCNA (amino acids 119-133) was replaced with the corresponding residues from human PCNA suggests that other residues in this loop are also involved directly in binding pol δ. Alternatively, it is possible that loop residues other than 123 and 126–128 play a secondary or indirect (e.g., conformational) role in positioning crucial amino acids so as to optimize their direct binding to pol δ.

In this context, we would like to call attention to the fact that at relatively low concentrations, dr119-133h dPCNA is considerably less effective than wild-type human PCNA in stimulating the activity of calf thymus pol δ; at higher concentrations, dr119-133h dPCNA and wild-type human PCNA stimulate calf thymus pol δ similarly. This implies complex protein-protein interactions between PCNA and pol δ such that biochemical properties recorded in dilute solutions *in vitro *may not accurately predict properties manifest at much different and generally much higher intranuclear concentrations present *in vivo*. Alternatively, PCNA must be present as a trimer (three-subunit ring) in order to function. Since the equilibrium among monomer, dimer and trimer was shown to depend on PCNA protein concentration [33], it is certainly possible that the difference observed between dr119-133h dPCNA and wild-type human PCNA actually reflects differences in the K_eq _for PCNA multimerization. These two possibilities, concerning both complicated pol δ•PCNA interactions and PCNA multimerization, are not mutually exclusive.

Similarly, the fact that replacement of the entire interdomain connector loop of *Drosophila *PCNA (amino acids 119-133) with the corresponding residues from human PCNA did not result in a molecule as effective in stimulating calf thymus pol δ as human PCNA suggests that regions other than the interdomain connector loop are important for pol δ-stimulation. Our data do not address the question of whether these putative "other regions" affect pol δ directly (e.g., like the interdomain loop) or indirectly (e.g., through conformational effects on other regions of the molecule that do bind pol δ directly). Additional mutagenesis studies may shed light on this issue. For example, based on experiments of others, it seems likely that the extreme C-terminus of PCNA also interacts directly with pol δ (see [23,34-36]). Hence it may be of interest to perform similar mutagenesis experiments to those reported here, focusing instead on the C-terminal region of *Drosophila *PCNA, rather than the interdomain connector loop.

We think it should also be noted that both Oku et al. [35] and Ola et al. [36] prepared hybrid proteins between human and *S. cerevisiae *PCNA. As in our studies, Ola et al. [36] found that regions other than the interdomain connector loop of PCNA were important for interaction with pol δ. These authors suggested that additional interacting regions were likely to exist both in the PCNA C-terminus and N-terminus.

It may also be of interest to prepare double-mutants, first in the interdomain connector loop of *Drosophila *PCNA, thereby allowing efficient *in vitro *function with purified calf thymus pol δ, and then elsewhere in the PCNA molecule corresponding to interesting sites defined phenotypically by *in vivo *genetic studies of others. For example, it might be possible to determine if particular *mus*209 mutations leading to enhanced mutagen sensitivity among affected organisms (see [37] and references therein) alter any functional interactions between PCNA and pol δ *in vitro*. Results of such studies could lead to novel biochemical insights regarding the mechanism(s) by which point mutations in the *Drosophila *PCNA gene lead to enhanced mutagen sensitivity among animals bearing these mutations.

The strategy taken here will presumably allow study of interactions between PCNA and other proteins with which it interacts. In this context, we think it important to note that partial effects on pol δ-stimulation have been recorded. This suggests that our methodology will also allow detection of partial rather than complete effects on the binding of other proteins. Interactions between PCNA and many of the molecules with which it interacts have recently been mapped [23] and for example, one might immediately compare interactions between several mammalian proteins (e.g., human RF-C, DNA ligase I, FEN I and/or p21) and both various wild-type and mutant PCNA molecules described in this paper. Functional (e.g., effects on pol δ activity) as well as direct binding measurements may be made. As with PCNA•pol δ interactions, it may ultimately be feasible to correlate interesting PCNA molecules defined phenotypically using genetic analyses performed in living animals and biochemical studies of specific PCNA•protein binding. For example, do mutagen sensitive *mus*209 animals bear mutations in a region of PCNA responsible for MSH binding? Both MSH3 and MSH6 were reported to possess a consensus motif for binding to the interdomain connector loop of PCNA [38].

Finally, we think it important to note that pol δ has most recently been reported to contain at least four subunits (see e.g., [39,40]) yet all experiments performed here were with the two-subunit form of the enzyme purified from calf thymus. We and others have shown that the larger subunit, p125, is catalytic while the smaller, p50, does not seem to contact the DNA closely (see e.g, [41]), but instead, is required for processivity-stimulation by PCNA (e.g., see [42]) to which it apparently binds. It is also clear that PCNA binds to what has been termed, the third pol δ subunit, p68 or p66 in mammalian systems [39,43,44], Cdc27p in *S. pombe *[40] and Pol32p in *S. cerevisiae *[45,46]. Clearly the physiologically important interaction between PCNA (either mutant or wild-type) and this third pol δ subunit was omitted from our analyses, but could markedly affect any or all of the responses of polymerase to PCNA that we reported here.

## Conclusions

Through our experiments, we showed that *Drosophila *PCNA could be "humanized" and that "humanization" (mutation of key *Drosophila *residues to human ones) increased effects on mammalian pol δ. The highly purified two-subunit form of pol δ was used for all of our studies. It is possible, though we think it unlikely, that different conclusions would be reached if a different form of pol δ (three-or four-subunit) was used. Nevertheless two of the effects we observed could be considered beneficial. They were enhancement of polymerase activity and processivity. A third effect seems likely to be detrimental, at least over the long term, that is increased synthesis opposite and beyond a chemically defined template base lesion (TLS). Our data suggest that all three of these effects result from enhancement of PCNA-dependent stability of the pol δ•PCNA•template-primer complex. In other words, in the range that we have studied, the more tightly pol δ binds to DNA, the greater its activity, the greater its processivity, but also the more likely it is to catalyze TLS. Our results provide an explicit approach to correlate *in vivo *genetic studies with rigorous *in vitro *biochemistry.

## Methods

### Materials

Unlabeled deoxyribonucleoside triphosphates (dNTPs) were from Boehringer-Mannheim; [α-^32^P]ATP and [α-^32^P]dTTP were from Amersham Corp. *E. coli *DNA polymerase I Klenow fragment without 3'-5' exonuclease activity (exo-), was expressed and purified according to standard protocols [47]. Terminal deoxynucleotidyl transferase (TdT) was from Sigma. Micrococcal nuclease was from Boehringer-Mannheim. Pfu DNA polymerase was from Stratagene. Ni^2+^-IDA Sepharose was from Pharmacia (Piscataway, NJ). Acrylamide and methylene bis-acrylamide were from Eastman Organic Chemicals and for protein SDS-PAGE, were further purified by adsorption of impurities to activated charcoal. For PAGE of nucleic acids, they were purified by adsorption to an ion exchange resin. All other materials were of reagent grade and were used without additional purification.

### Proteins

PCNA was purified to apparent homogeneity from calf thymus [17] as was pol δ [24,48]. Human PCNA cDNA was cloned into a bacterial expression vector and human PCNA was purified from an *E. coli *extract, also to apparent homogeneity [18]. *D. melanogaster *PCNA was purified to apparent homogeneity identically after bacterial expression [13]. A his-tag was added to the NH_2_-termini of both human and *Drosophila *PCNA by cDNA insertion into pQE30 (Qiagen, Valencia, CA) using *Bam*H1 and *Hind*III restriction endonuclease sites.

### Nucleic acids

Templates and primers, all of defined sequence, were synthesized conventionally by Dr. F. Johnson and colleagues (Stony Brook). Before use, they were purified by standard denaturing PAGE [49]. All other DNA manipulations were performed according to standard techniques [49].

### Methods

Much of the methodology was described in detail previously [12,19,20,24,29,32,41,50,51]. SDS-PAGE was according to Laemmli [52] as modified [53] on minigels or as reported previously [54]. For immunoblots, proteins were transferred electrophoretically to nitrocellulose [55] and resulting replicas were probed with antibodies. Reactivity was visualized colorimetrically [56] with alkaline phosphatase-conjugated goat anti-IgG antibodies [57,58] and a one-solution phosphatase substrate (Kirkegaard and Perry, Gaithersburg, MD). Immunologic detection of human PCNA was with mouse monoclonal antibody (mAb) PC10 (Oncogene Sciences, Uniondale, NY). Detection of *Drosophila *PCNA was with affinity purified polyclonal rabbit anti-*Drosophila *PCNA antibodies [12]. Restriction endonucleases were from Boehringer (Indianapolis, IN) and were used according to the vendor's instructions. DNA sequencing performed in both directions was according to Sanger et al. [59] using a fluorescence-based method and an ABI 373 (Applied Biosystems, Foster City, CA) automated DNA sequencer.

### Site-directed mutagenesis of *Drosophila* PCNA

Site-directed mutagenesis of NH_2_-terminally his-tagged *Drosophila *PCNA was performed exactly as described [20] to generate either the Q123V protein or chimeric molecules containing the entire *Drosophila *PCNA sequence except for amino acids 119-133 which were replaced by the corresponding residues from human PCNA.

### Purification of his-tagged PCNA

Purification of his-tagged PCNA to apparent homogeneity was performed exactly as previously described [20]. Characterization was by SDS-PAGE (Fig. 2) and immunoblot analysis.

### DNA polymerase δ incubations

Assays of pol δ on synthetic oligonucleotide template-primers were performed essentially as previously described [24]. Primers were 5' end-labeled with T4 polynucleotide kinase in the presence of [γ-^32^P]ATP. Afterward, labeled primer was annealed to an unlabeled template. The standard reaction mixture for pol δ contained 40 mM Bis-Tris, pH 6.7, 6 mM MgCl_2_, 1 mM dithiothreitol, 10% glycerol and 40 μg/ml bovine serum albumin. Additional details are provided in the figure legends. Incubations were terminated by addition of standard stop solution and aliquots were subjected to 12% PAGE in the presence of 7 M urea and 15% formamide. After electrophoresis, gels were subjected to autoradiography and/or Molecular Dynamics 445 SI PhosphorImager analyses.

### Pol δ processivity

Processivity was evaluated qualitatively using (dA)_~500 _annealed to (dT)_12–18 _(both from Pharmacia) in a final volume of 5 μl containing 6 nmol poly(dA) (nucleotide), 0.2 nmol (dT)_12–18 _(nucleotide), 10 μM dTTP, 100 μCi [α-^32^P]dTTP, 40 mM Bis-Tris, pH 6.7, 6 mM MgCl, 1 mM dithiothreitol, 10% glycerol, 40 μg/ml bovine serum albumin, 10 ng of highly purified pol δ and various quantities of different PCNA samples as indicated. Assays were for 5 min at room temperature and were stopped by addition of standard PAGE stop solution and PAGE in the presence of 7 M urea. After electrophoresis, gels were subjected to autoradiography and/or Molecular Dynamics 445 SI PhosphorImager analyses.

### Nondenaturing PAGE band mobility shift assays

Nondenaturing PAGE band mobility shift assays were performed essentially as previously described [32] but without MgCl_2 _and otherwise as detailed in the figure legend. EDTA was included in each incubation and in the gel electrophoresis buffer at a final concentration of 3 mM.

## Authors' contributions

DJuM performed all enzymologic and mobility shift assays with DNA polymerase δ in combination with both wild-type and various mutant PCNA molecules. He also designed, engineered and characterized all recombinant PCNA molecules. DJuM expressed several recombinant proteins in bacteria and purified them. Finally, he participated in DNA polymerase purification and drafted the original manuscript. MM expressed some recombinant proteins in bacteria and purified them. She also purified and characterized most DNA polymerase substrates. HM participated in DNA polymerase purification and manuscript preparation. PAF advised DJuM on execution and interpretation of experiments and assisted both in figure design and all other aspects of manuscript preparation. All authors read and approved the final manuscript.
